# Adhoc electromagnetic compatibility testing of non-implantable medical devices and radio frequency identification

**DOI:** 10.1186/1475-925X-12-71

**Published:** 2013-07-11

**Authors:** Seth J Seidman, Joshua W Guag

**Affiliations:** 1 10903 New Hampshire Ave, White Oak Bldg 62 Room 1134, Silver Spring, MD 20993, USA

**Keywords:** Electromagnetic compatibility, Electromagnetic interference, EMC, EMI, Radio-frequency identification, RFID, Medical device

## Abstract

**Background:**

The use of radiofrequency identification (RFID) in healthcare is increasing and concerns for electromagnetic compatibility (EMC) pose one of the biggest obstacles for widespread adoption. Numerous studies have documented that RFID can interfere with medical devices. The majority of past studies have concentrated on implantable medical devices such as implantable pacemakers and implantable cardioverter defibrillators (ICDs). This study examined EMC between RFID systems and non-implantable medical devices.

**Methods:**

Medical devices were exposed to 19 different RFID readers and one RFID active tag. The RFID systems used covered 5 different frequency bands: 125–134 kHz (low frequency (LF)); 13.56 MHz (high frequency (HF)); 433 MHz; 915 MHz (ultra high frequency (UHF])) and 2.4 GHz. We tested three syringe pumps, three infusion pumps, four automatic external defibrillators (AEDs), and one ventilator. The testing procedure is modified from American National Standards Institute (ANSI) C63.18, Recommended Practice for an On-Site, Ad Hoc Test Method for Estimating Radiated Electromagnetic Immunity of Medical Devices to Specific Radio-Frequency Transmitters.

**Results:**

For syringe pumps, we observed electromagnetic interference (EMI) during 13 of 60 experiments (22%) at a maximum distance of 59 cm. For infusion pumps, we observed EMI during 10 of 60 experiments (17%) at a maximum distance of 136 cm. For AEDs, we observed EMI during 18 of 75 experiments (24%) at a maximum distance of 51 cm. The majority of the EMI observed was classified as probably clinically significant or left the device inoperable. No EMI was observed for all medical devices tested during exposure to 433 MHz (two readers, one active tag) or 2.4 GHz RFID (two readers).

**Conclusion:**

Testing confirms that RFID has the ability to interfere with critical medical equipment. Hospital staff should be aware of the potential for medical device EMI caused by RFID systems and should be encouraged to perform on-site RF immunity tests prior to RFID system deployment or prior to placing new medical devices in an RFID environment. The methods presented in this paper are time-consuming and burdensome and suggest the need for standard test methods for assessing the immunity of medical devices to RFID systems.

## Background

The use of radiofrequency identification (RFID) in healthcare is increasing and concerns for electromagnetic compatibility (EMC) pose one of the biggest obstacles for widespread adoption. Numerous studies have documented that RFID can interfere with medical devices [1-7] while [8,9] reported no electromagnetic interference (EMI). The majority of past studies have concentrated on implantable medical devices such as implantable pacemakers and implantable cardioverter defibrillators (ICDs). This study examined EMC between RFID systems and non-implantable medical devices.

The EMI mechanisms in most non-implantable medical devices are different from those of the implantable devices that we tested previously. Pacemakers and ICDs both have electrophysiological sensing capabilities, and EMI to these devices from RFID is mainly attributed to the medical devices inappropriately sensing the RFID output as an intrinsic cardiac signal. EMI to non-sensing devices occurs when a source (i.e., an RFID reader) couples energy to a victim’s internal electronics. This coupling path could be inductive, capacitive, or electromagnetic.

RFID is an identification system that is used to locate, identify, and track objects. The system consists of a transponder (tag) and an interrogator (reader). RFID readers read and write information to tags using radiofrequency (RF) energy. The tags can be attached to any physical object, including medicine containers, hospital room equipment, vehicles, medical devices, envelopes, packages and even animals and humans. Readers physically range from large portal antennas to desktop pad workstations to small handheld portable readers. Tags that transmit RF and contain batteries (active tags) must be at least as large as the battery, while tags that backscatter RF energy (passive tags) can be small enough to fit inside a pill. The range for reading RFID tags is constrained by many factors, including the output power and the carrier frequency of the reader. More in depth information about RFID can be found in Finkenzeller [10].

A detailed investigation into FDA adverse event reports revealed 74 records that contain the word “RFID” between 1991 and 2013. However, it is clear that the majority (if not all) of these records are not related to EMI due to RFID exposure. In our expert opinion only 2 records (MDR Report #6000153-2007-02403 and #2936485-2005-00068) have the possibility to be related to EMI. However we cannot conclude anything definite due to a lack of detailed information in the 2 reports. So while we believe this research is proactive, we understand that EMC issues are vastly underreported (due to difficulty in diagnosing), and real world problems may already exist.

The work described here is important due to the proliferation of RFID in the healthcare environment. Ad hoc EMC experiments are necessary to diagnose potential problems and identify possible solutions so that RFID and medical devices can coexist. Results from these experiments will assist in determining the risk for each type of medical device and data will be used in the development and validation of an RFID simulator. An RFID simulator will speed up future testing and be a key component in the development of applicable test methods and standards.

## Methods

Medical devices were exposed to 19 different RFID readers and one RFID active tag. The RFID readers used covered 5 different frequency bands: 125–134 KHz (low frequency (LF)); 13.56 MHz (high frequency (HF)); 433 MHz; 915 MHz (ultra high frequency (UHF)) and 2.4 GHz. The RFID active tag operated at 433 MHz. We tested three syringe pumps, three infusion pumps, four automatic external defibrillators (AEDs), and one ventilator.

RFID field levels were measured for each RFID system within a plane with 2.5 cm separation distance. The vector magnitude RMS magnetic field strength was measured using magnetic field probe (Electric Research and Management, model 1709.001) for 125–134 kHz RFID readers and using magnetic field probe (Speag, H3DV7) for 13.56 MHz RFID readers. The vector magnitude RMS electric field was measured using an electric field probe (Narda, SRM 3-Axis E-Field Antenna) for 13.56 MHz RFID readers and an isotropic electric field probe (ETS Lindgren, Model HI-6105) for RFID systems at 433, 915, and 2450 MHz. We captured the maximum field strength value by performing a peak hold at each measurement point for a duration of 15 s.

The test was performed in a fully anechoic chamber to minimize uncontrolled factors such as reflections and outside RF. The medical device under test (DUT) in our experiment was placed on a non-conductive table and configured for its normal operation. For AED testing, a patient simulator (Netech, Delta 1500) was used and shielded from the RFID exposure. If the device had sensing capability, the sensors of the DUT were extended straight back as much as possible. For AED testing we tested the device with both a normal sinus rhythm signal and with a ventricular fibrillation signal from our patient simulator. For other DUT types typical operating modes were tested.

We programmed the RFID readers to transmit at their maximum output power and continuously poll for tags via the inventory command. The RFID reader antenna was mounted on a non-conductive frame and placed next to the DUT. The RFID reader antenna was centered to one side of the DUT being exposed. We exposed the DUT to a RFID reader antenna at 1 cm separation distance. The DUT was exposed to the RFID reader for one complete cycle, which consisted of the device boot-up, programming the device, and one complete operation cycle of the device (complete delivery of therapy for pumps or complete analyzing of heart beat for AEDs). The operation time ranged from 12 s to 1 minute, depending on the device. For devices that have a short, repeating operational cycle, such as a ventilator, the exposure time was 15 s after it was turned on and programmed.

**Figure 1:**
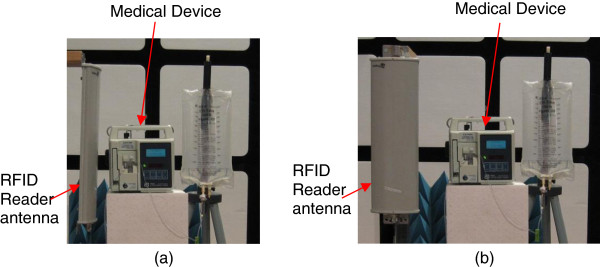
RFID reader orientations tested: (a) horizontal orientation; (b) vertical orientation.

If EMI was observed, we moved the RFID reader antenna away in 5 cm increments and the test was repeated until the DUT changed operation. Next we moved the RFID reader antenna closer to the DUT in 1 cm increments until the original EMI repeated. This distance is the threshold distance for EMI. During the process, the DUT was turned off and restarted for each step to eliminate any potentially non-transient effects. The procedure was repeated for all five exposed surfaces of the DUT (including sensor cables) and two different orientations (Figure 1) for each RFID reader antenna.

The testing procedure is similar to the research testing clause of ANSI C63.18, Recommended Practice for an On-Site, Ad Hoc Test Method for Estimating Radiated Electromagnetic Immunity of Medical Devices to Specific Radio-Frequency Transmitters [11] except our testing began at the device surface and moved further away if EMI was observed.

One experiment was defined as all testing performed on one medical device by one RFID system. This includes testing for all five surfaces of the DUT with both of the selected RFID orientations across all separation distances. While we realize that 1 cm separation distance is very uncommon in most medical applications it was the authors’ desire to test to the minimum separation distance because each RFID use case will be different. There are RFID medical applications in which a 1 cm separation distance is feasible. Test data at 1 cm can be valuable information for such RFID implementations. For most applications 1 cm may not be possible, and test data at this distance may have little clinical relevance.

## Results

We tested three syringe pumps, three infusion pumps, one ventilator, and four AEDs. For all medical devices, we observed EMI in 19% (41 of 215) of the experiments. The maximum EMI threshold distance was 136 cm. No EMI was observed for all medical devices tested during exposure to 433 MHz (two readers, one active tag) or 2.4 GHz RFID (two readers). Additionally no EMI was observed for the ventilator during exposure to any RFID system. We observed the following transient effects: false alarms, disrupted displays, device failure to turn on, inappropriate delivery of cardiac therapy, failure to deliver cardiac therapy and other less clinically significant effects. Additionally, one AED was permanently damaged during exposure to RFID Reader 17. During exposure, the AED emitted audible noise, failed to perform analysis, and failed to deliver therapy. We were unable to download the data log from AED after the exposure, and the exact failure type could not be determined. After exposure, the AED was not able to analyze or deliver therapy. EMI types were classified as Probably Not Clinically Significant, Device Inoperable, or Probably Clinically Significant. See Table 1 for a complete list of observed EMI types, their descriptions, and classification. Note that the classification scheme does not comment on the clinical significance of a device being inoperable from exposure to RFID. Both the authors and medical officers within the FDA could not make an informed determination about the clinical significance of a device being inoperable without knowing more information about the patient and the particulars of how the device was being used. We did our best using common sense judgment to make a determination when other EMI was observed.

**Table 1:** List of EMI observed

***Description of medical device EMI***	***EMI classification***
Device takes unusually long time to turn on	
Minor malfunction of display. The display is still comprehensible.	probably not
Audible noise from the device (e.g. speaker hum or buzz)	clinically
Device inappropriately alarmed	significant
Unable to turn the device on	
Occasionally unable to turn the device on	
Device permanently damaged	
Unable to turn off the device once turned on	
Unable to program the device	device
Unable to pump	inoperable
Major malfunction of display. The display is incomprehensible.	
AED could not detect patient	
AED sensed false motion	
AED inappropriately did not advise shock for VFIB (Ventricular fibrillation) patient input	
Occasionally, AED inappropriately did not advise shock for VFIB patient input	probably
AED inappropriately advised shock for NSR (Normal Sinus Rhythm) patient input	clinically
AED advised shock for VFIB patient input, but failed to deliver. Returned to analysis mode	significant

**Figure 2:**
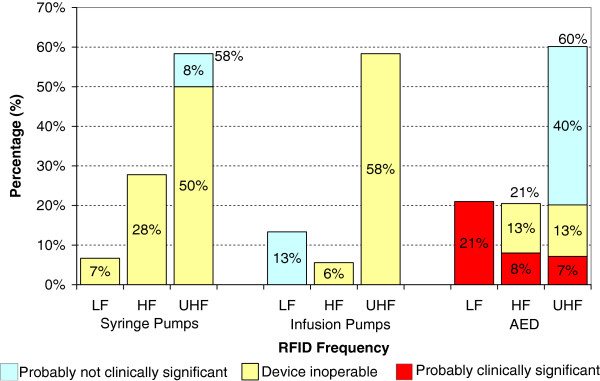
**Percentage of medical devices experiencing EMI for each device category at different RFID frequency ranges.** LF = 134 kHz, HF = 13.56 MHz, UHF = 915 MHz. No EMI was observed at 433 MHz or 2.45 GHz. No EMI was observed for the one a ventilator tested.

**Figure 3:**
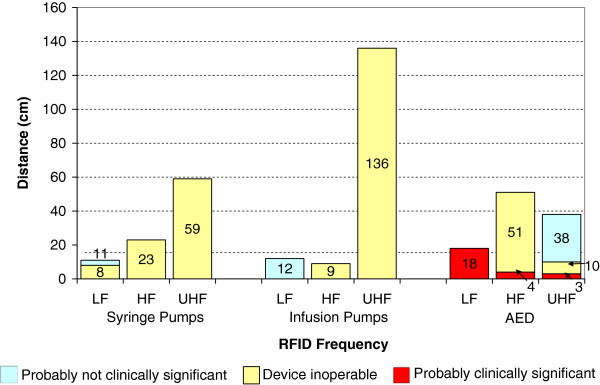
**Maximum distances where EMI was observed for each device category at different RFID carrier frequencies.** LF = 134 kHz, HF = 13.56 MHz, UHF = 915 MHz. No EMI was observed at 433 MHz or 2.45 GHz. No EMI was observed for the one ventilator tested.

For syringe pumps, we observed EMI during 13 of 60 experiments (22%) at a maximum EMI threshold distance of 59 cm. Of the 13 experiments where interference was observed 12 made the device inoperable. For infusion pumps, we observed EMI during 10 of 60 experiments (17%) at a maximum EMI threshold distance of 136 cm. Of the 10 experiments where interference was observed, 8 made the device inoperable. For AEDs, we observed EMI during 18 of 75 experiments (24%) at a maximum EMI threshold distance of 51 cm. Of the 18 experiments where interference was observed, 7 were classified as probably clinically significant and 5 made the device inoperable. All effects except the permanently damaged AED were transient effects; once the RFID exposure was removed the device returned to normal operation. See Tables 2, 3 and 4 for all data on syringe pumps, infusion pumps, and AEDs. RFID system characteristics along with EMI occurrence data are shown in Table 5. The percentage of EMI observed and the maximum distance where EMI was observed for each medical device is presented for each RFID frequency (Figures 2 and 3).

**Table 2:** Test data for syringe pumps

		**Probably not clinically significant**	**Device inoperable**	**Probably clinically significant**	**All experiments**
Syringe Pump #1 (1994)	Occurrences of EMI	4	10	0	10 (50%)
	Max Distance of EMI (cm)	23	40	-	40
	RFID Reader Code causing EMI	5,8,10,11	5,7-11,15-18	N/A	5,7-11,15-18
Syringe Pump #2 (2004)	Occurrences of EMI	3	2	0	3 (15%)
	Max Distance of EMI (cm)	59	59	-	59
	RFID Reader Code causing EMI	15-17	15,17	N/A	15-17
Syringe Pump #3 (2002)	Occurrences of EMI	0	0	0	0 (0%)
	Max Distance of EMI (cm)	-	-	-	-
	RFID Reader Code causing EMI	N/A	N/A	N/A	N/A

The EMI Occurrences row represents the number of times EMI occurred during a test (n = 20). One experiment was defined as all testing performed on one medical device with one RFID system. Therefore the All Experiments column indicates whether any EMI occurred for a particular RFID system and does not indicate whether multiple effects were caused by the same RFID system. Production years for devices tested in parenthesis.

**Table 3:** Test data for infusion pumps

		**Probably not clinically significant**	**Device in operable**	**Probably significant**	**All experiments**
Infusion Pump #1 (1995)	Occurrences of EMI	0	4	0	4 (20%)
	Max Distance of EMI (cm)	-	68	-	68
	RFID Reader Code causing EMI	N/A	8,15,17,18	N/A	8,15,17,18
Infusion Pump #2 (1996)	Occurrences of EMI	1	4	0	5 (25%)
	Max Distance of EMI (cm)	9	136	-	136
	RFID Reader Code causing EMI	5	15-18	N/A	5,15-18
Infusion Pump #3 (2002)	Occurrences of EMI	1	0	0	1 (5%)
	Max Distance of EMI (cm)	12	-	-	12
	RFID Reader Code causing EMI	5	N/A	N/A	5

The EMI Occurrences row represents the number of times EMI occurred during a test (n = 20). One experiment was defined as all testing performed on one medical device with one RFID system. Therefore the All Experiments column indicates whether any EMI occurred for a particular RFID system and does not indicate whether multiple effects were caused by the same RFID system. Production years for devices tested in parenthesis.

**Table 4:** Test data for AEDs

		**Probably not clinically significant**	**Device in operable**	**Probably clinically significant**	**All experiments**
AED #1 (2008)	Occurrences of EMI	3	0	0	3 (15%)
	Max Distance of EMI (cm)	21	-	-	21
	RFID Reader Code causing EMI	15,17,18	N/A	N/A	15,17,18
AED #2 (2002)	Occurrences of EMI	1	3	2	3 (15%)
	Max Distance of EMI (cm)	11	10	18	18
	RFID Reader Code causing EMI	17	3,5,17	3,5	3,5,17
AED #3 (2009)	Occurrences of EMI	2	4	4	8 (40%)
	Max Distance of EMI (cm)	14	51	18	51
	RFID Reader Code causing EMI	15,17	7,8,10,11	3,5,7,17	3,5,7,8,10,11,15,17
AED #4 (2004)	Occurrences of EMI	2	1	1	4 (20%)
	Max Distance of EMI (cm)	38	0	1	38
	RFID Reader Code causing EMI	15,16	17	10	10,15-17

The EMI Occurrences row represents the number of times EMI occurred during a test (n = 20). One experiment was defined as all testing performed on one medical device with one RFID system. Therefore the All Experiments column indicates whether any EMI occurred for a particular RFID system and does not indicate whether multiple effects were caused by the same RFID system. Production years for devices tested in parenthesis.

**Table 5:** RFID system characteristics

**RFID reader code**	**Emitter antenna dimensions (cm)**	**Carrier frequency (MHz)**	**Pulse repetition rate (Hz)**	**Duty factor**	**Pulse width (ms)**	**Max field intensity (A/m RMS) @ 2**.**5 cm**	**Max field intensity (V/m RMS)**	**Governing ISO standard**	**Occurrences of EMI**
1	Rectangular Loop 3.5 × 4.5 × 0.5	0.125	CW				--	--	0
2	Rectangular Loop 114 × 66 × 6.3	0.125	NA			13	--	--	0
3	Rectangular Loop 85 × 50 × 5	0.134	14.3	0.72	49.9	93	--	11785	2
4	Rectangular Loop 85 × 50 × 5	0.134	CW			47	--	--	0
5	Rectangular Loop 20 × 20 × 2.5	0.134	10.6	0.54	50.3	119	--	11785	4
6	Rectangular Loop 31 × 31 × 2.8	13.56	10.9	0.11	10.3	1	16	18000-3 mode 1	0
7	Rectangular Loop 20 × 20 × 0.8	13.56	4.0	0.13	31.9	1	60	18000-3 mode 1	2
8	Rectangular Loop 31 × 31 × 2.8	13.56	11.1	1.0	90.0	6	67	18000-3 mode 1	3
9	Patch 2.3 × 2.5 × 0.1	13.56	2.6	0.95	362.6	2		18000-3	1
10	Patch 21 × 32 × 1.2	13.56	1.0	0.04	50.5	3	378	18000-3 mode 2	3
11	Handheld 19 × 11 × 7.8	13.56	3.5	0.92	264.0	4	71	18000-3 mode 1	2
12	Stick length 19.8 × diameter 1.4	433	NA			--	0*	--	0
13	Patch 38 × 36.5 × 1.5	433	NA			--	0*	--	0
14	Patch 15.7 × 5.5 × 3	433	NA			--	0*	18000-7	0
15	Patch 31 × 31 × 4.8	915	56.1 k	0.76	0.01	--	59	18000-6B	7
16	Patch 48.5 × 16 × 5	915	NA			--	33	18000-6B	4
17	Patch 21 × 21 × 3.5	915	NA			--	89	18000-6	8
18	Patch 22.5 × 21 × 5	915	NA			--	73	18000-6C	4
19	Stick length 10.5 diameter 0.9	2450	NA			--	1	--	0
20	Stick length 11 diameter 0.8	2450	897	0.16	0.18	--	2	--	0

This table describes all RFID equipment tested. It describes the carrier frequency, magnetic field strength, electric field strength, the applicable ISO RFID standard, and the number of occurrences of EMI. *RFID Readers 12 and 13 only receive 433 MHz (no transmit capability). RFID #14 is the active tag.

## Discussion

EMI was most prevalent during exposure to 915 MHz RFID readers. This is consistent with the findings of most of the research in this area [3,6,7]. This is not surprising because the radiated RF immunity test level for medical devices that are life-supporting specified by the International standard IEC 60601-1-2 [12] for EMC of medical electrical equipment and systems at 915 MHz is 10 V/m. (The radiated RF immunity test level for non-life-supporting equipment and systems is 3 V/m.) The maximum measured electric field strength from our 915 MHz RFID readers was 89 V/m at 2.5 cm. So at 2.5 cm our experiment is exposing medical devices to field strengths 9 to 30 times higher than those to which they would be expected to have been tested. All devices we tested were life-supporting devices presumably tested at 10 V/m. IEC 60601-1-2 also provides guidance to keep a minimum separation distance between the medical device and RF communication equipment at 915 MHz. Our readers ranged from 1 – 4 W and the recommended separation distance ranges between 70 and 140 cm. Only Infusion Pump #2 exhibited EMI at a distance greater than the recommended separation distance.

No EMI was observed for all medical devices tested during exposure to 433 MHz (two readers, one active tag) or 2.4 GHz RFID (two readers) where the measured electric field strengths were all below the 3 V/m that medical equipment that is not life-supporting is tested to per IEC 60601-1-2. Similar comparisons are not possible when discussing 125–134 kHz and 13.56 MHz systems because IEC 60601-1-2 does not require medical devices to be tested to radiated magnetic fields in those frequency ranges.

It is clear that more testing is necessary to fully understand the medical device risks associated from RFID exposure. Unfortunately medical device testing with all RFID frequencies performed according to ANSI C63.18 is extremely burdensome and time consuming due to the number of RFID systems (and modes of operation), the different medical device modes of operation, and the combinations of test orientations and separation distances. The data recorded from these experiments will assist in the development of RFID simulators capable of replacing commercial RFID systems for EMC testing. The RFID simulators will need to be able to reproduce the same signals as the commercial readers but will use a standard antenna source. Separation distances can be simulated by adjusting the output power. This will cut testing time per medical device from 8 hours to about 30 minutes. Medical device manufacturers could use these simulators as a way to show that their devices are immune to RFID emissions.

### Study limitations

Although all medical devices were in good working order, the devices were previously used and it is possible that they might have some unknown prior damage. Additionally, we were not able to test multiple devices of the same make and model which could lead to different results. More devices should be tested in the future to permit a better understanding of EMI mechanisms.

Our presence during the tests could have affected the electromagnetic field. This was minimized by performing tests in an anechoic chamber and by not placing our body between the RFID reader and the DUT. This is mostly of concern while testing medical devices that require programming, as we had to be near the device in order to program the DUT.

We do not believe EMI could be caused from the low output power of passive RFID tags. However, inclusion of 1 or more passive RFID tags in the system could have an effect on the RFID reader waveform. This effect could just as easily increase or decrease the chance of observing interference. As this testing was already extremely burdensome and time consuming we did not include passive tags in our system exposure.

The tests were performed with each RFID reader antenna centered at the selected side of the DUT and only two orientations of the RFID reader antenna were used due to time constraints. Therefore, any EMI that could be caused by off-center exposures to the DUT or by other orientations of the RFID devices were neglected.

EMI could be observed outside the dwell time that was specified in our test protocol.

## Conclusions

Testing confirmed that RFID systems have the ability to interfere with critical medical equipment. The results from this testing indicate that 915 MHz RFID readers can be the most problematic for medical devices. No EMI was observed for all medical devices tested during exposure to 433 MHz (two readers, one active tag) or 2.4 GHz RFID (two readers). It is clear that more testing is necessary but current methods are extremely burdensome and time consuming. Current medical device EMC standards need to be updated to include field levels and signals that represent actual RFID systems. Moving forward, we hope to develop RFID simulators that could be the basis of an RFID EMC test standard. This will help the medical device community to both identify and mitigate potential EMI from RFID. Hospital staff should be aware of potential medical device EMI from RFID systems and are encouraged to perform on-site EMC tests prior to RFID deployment or prior to placing new medical devices in an RFID environment.

## Abbreviations

RFID: Radiofrequency identification; EMC: Electromagnetic compatibility; ICDs: Implantable cardioverter defibrillators; LF: Low frequency; HF: High frequency; UHF: Ultra high frequency; AEDs: Automatic external defibrillators; ANSI: American National Standards Institute; EMI: Electromagnetic interference; RF: Radiofrequency; DUT: Device under test.

## Competing interests

The authors declare that they have no competing interests.

## Authors' contributions

SJS and JWG have made substantial contributions to the conception and design, acquisition, analysis and interpretation of data. Both authors were involved in drafting the manuscript. Both authors read and approved the final manuscript.
